# Anatomical study for elucidating the stabilization mechanism in the trapeziometacarpal joint

**DOI:** 10.1038/s41598-022-25355-3

**Published:** 2022-12-01

**Authors:** Mio Norose, Akimoto Nimura, Masahiro Tsutsumi, Koji Fujita, Atsushi Okawa, Keiichi Akita

**Affiliations:** 1 Department of Clinical Anatomy, Graduate School of Medical and Dental Sciences, Tokyo Medical and Dental University, Tokyo, Japan; 2 Department of Orthopaedic and Spinal Surgery, Graduate School of Medical and Dental Sciences, Tokyo Medical and Dental University, Tokyo, Japan; 3 Department of Functional Joint Anatomy, Graduate School of Medical and Dental Sciences, Tokyo Medical and Dental University, 1-5-45 Yushima, Bunkyo-Ku, Tokyo, 113-8519 Japan; 4 Inclusive Medical Science Research Institute, Morinomiya University of Medical Sciences, Osaka, Japan

**Keywords:** Ligaments, Muscle, Tendons

## Abstract

To determine the pathogenesis of trapeziometacarpal (TMC) joint instability, which leads to osteoarthritis, we investigated the anatomical relationships among the surrounding ligaments, muscles (first dorsal interosseous [FDI] and opponens pollicis [OPP]), and joint capsule. We examined the bone morphology and cortical bone thickening in 25 cadaveric thumbs using micro-computed tomography and performed macroscopic and histological analyses. The dorsal trapezium had a tubercle with cortical bone thickening, corresponding to the attachment of the FDI aponeurosis intermingled with the joint capsule. Radially, the thin joint capsule was observed to underlie the muscular part of the OPP. Therefore, the dorsal ligaments, which have been previously considered static stabilizers, could be interpreted as parts of the capsuloaponeurotic complex consisting of the FDI aponeurosis and joint capsule. In the radial aspect, muscular OPP activation may be essential for TMC joint stabilization. Our findings may contribute to the appropriate management of TMC osteoarthritis.

## Introduction

The trapeziometacarpal joint (TMC joint) is a biconcave–convex saddle joint that is composed of the first metacarpal bone (1st MC) and the trapezium^1^. It allows a wide range of motion and multidirectional movement of the thumb^2,3^. Joint instability due to overuse or injury leads to TMC osteoarthritis, which results in pain, dysfunction of the joint, and dorsoradial subluxation of the 1st MC^4–6^. Stabilization of the TMC joint has been clarified by two mechanisms: static (with the surrounding ligaments) and dynamic (with the adjoining muscles)^7–13^. However, laxity and disruption of the dorsoradial ligaments have been assumed to be the pathogenic mechanisms underlying TMC joint instability^3,14,15^, which subsequently leads to TMC osteoarthritis and dorsoradial subluxation of the 1st MC^6,8,15^. Identification of a clear anatomical relationship between the static and dynamic stabilizing structures, particularly in the dorsoradial part of the TMC joint, would be helpful to determine the actual pathogenesis of TMC joint instability.

The dorsal ligaments and the anterior oblique ligament (AOL) have been described as important static stabilizers during pinching^7,9,10,16,17^. However, “ligaments” have been described as not being histologically distinguishable from the surrounding aponeuroses and joint capsule^18,19^. Recently, anatomical studies have reported that in joints such as the elbow^20,21^, hip^22^, and knee^23^, “ligaments” are interpreted as parts of the periarticular aponeurosis, which is believed to contribute to dynamic stabilization^20–24^. Although the ligaments adjoin the surrounding muscles in the TMC joint, the layered relationships between the ligaments and the surrounding elements, such as the muscle, aponeurosis, and joint capsule, have been rarely discussed.

We proposed that the ligaments of the TMC joint are part of the periarticular aponeuroses of the first dorsal interosseous (FDI), opponens pollicis (OPP), and joint capsule. Specifically, we hypothesized that the cortical bone, on which the tensile stress was loaded, and the joint capsule, which intermingled with the aponeurosis, were thicker than other areas. The aim of this study was to anatomically analyze the TMC joint based on the surrounding muscles and joint capsule rather than on the specific ligaments. This may help to elucidate the mechanism underlying the dynamic stability of the TMC joint based on anatomical findings, which could hold some clues to the improvement of TMC osteoarthritis management.

## Results

### Osseous surface morphology and cortical bone thickness of the trapezium and 1st MC determined using micro-computed tomography

**Figure 1:**
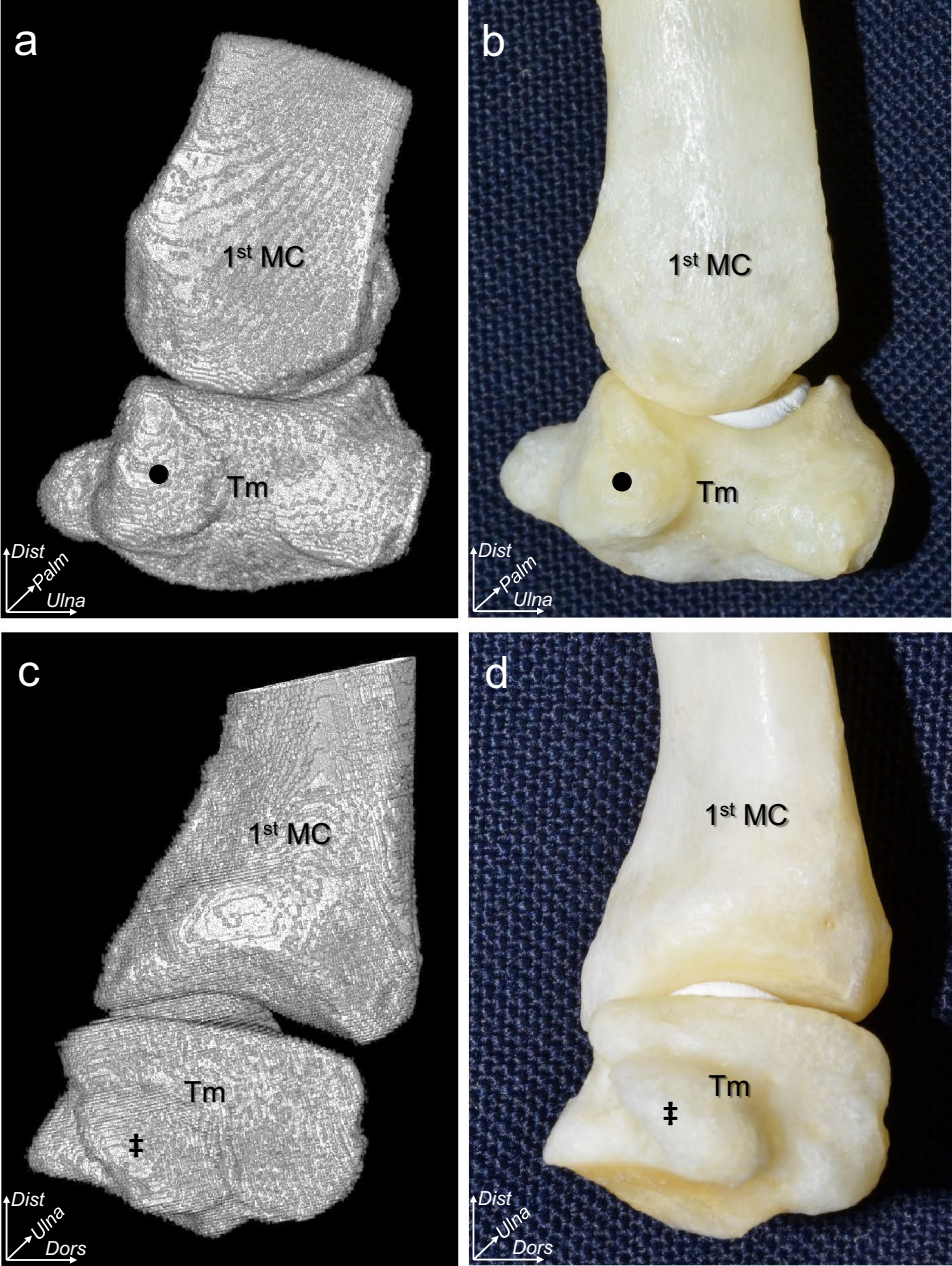
Bone morphology of the trapeziometacarpal joint. Three-dimensional (3D) computed tomography images and actual bone images of the dorsal (**a** and **b**) and radial aspects (**c** and **d**) of the trapezium (Tm) and the first metacarpal bones of the right thumb. (**a**) the Tm is prominent (black circle) on the radial side of the dorsal aspect. (**b**) the actual bone images of (**a**). (**c**,**d**) 3D images and actual bone images of the radial aspect of the trapeziometacarpal joint, respectively. The double-dagger shows the radial roof of the flexor carpi radialis tendon groove. *Dist*, distal; *Ulna*, ulnar; *Dors*, dorsal; *Palm*, palmar.

Micro-computed tomography (micro-CT) revealed a tubercle with a bony morphology on the radial side of the dorsal aspect of the trapezium (Fig. 1a), which was confirmed in chemically debrided bones (Fig. 1b). On the radial aspect, the trapezium had no characteristic bony morphology, except for the flexor carpi radialis tendon groove (Fig. 1c,d).

**Figure 2:**
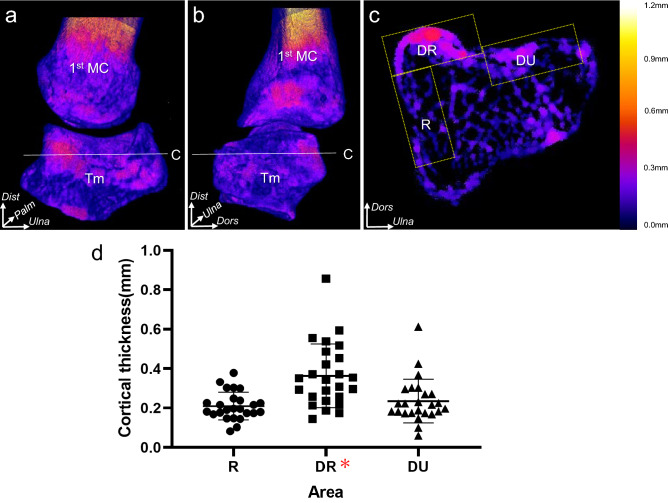
Evaluation of cortical bone thickening in the trapezium and the proximal part of the metacarpal bone. Mapping of the cortical thickening in the right trapeziometacarpal joint was performed after image processing. The thicker the cortical bone of the point, the brighter is the color of the point. (**a**,**b**) dorsal and radial aspects of three-dimensional cortical bone thickness images. (**c**) axial section at the level of the white line in (**a**) and (**b**). The cortical bone is divided into the radial side of the dorsal aspect (DR), ulnar side of the dorsal aspect (DU), and radial aspect (R) using equivalent rectangles, which are indicated by yellow dotted areas. The color bars (from black to navy blue, violet, red, orange, yellow, and white) indicate the cortical bone thickness distribution (mm). (**d**) scatterplot showing cortical bone thickness of the rectangles. Cortical bone thicknesses are presented as means and standard deviations. The mean cortical thickness of the DR (red asterisk) was significantly greater than the corresponding thicknesses of the DU and R (repeated-measures analysis of variance and Bonferroni *post-hoc* test, both P < 0.001). *Dist*, distal; *Ulna*, ulnar; *Dors*, dorsal; *Palm*, palmar.

Mapping of the cortical bone thickness using micro-CT data revealed that the cortical bone of the dorsoradial tubercle of the trapezium was brightly colored (Fig. 2a), and the radial aspect showed no brightly colored area (Fig. 2b), which indicated that the cortical bone of the dorsoradial tubercle of the trapezium was thicker than other areas. The mean cortical bone thickness was significantly greater at the radial side of the dorsal aspect of the trapezium (0.4 ± 0.2 mm, **DR** in Fig. 2c,d) than at the ulnar sides of the dorsal aspect (0.2 ± 0.1 mm, P = 0.00000092, **DU** in Fig. 2c,d) and the radial aspect (0.2 ± 0.1 mm, P = 0.000019, **R** in Fig. 2c,d) of the trapezium. In addition, we performed subgroup analyses on two sets of groups: age-based (those aged < 85 years and ≥ 85 years) and sex-based. The intergroup differences were significant for all parameters, except for those in the male group (Supplementary Table S1).

### Macroscopic analysis of the dorsal and radial aspects of the TMC joint

**Figure 3:**
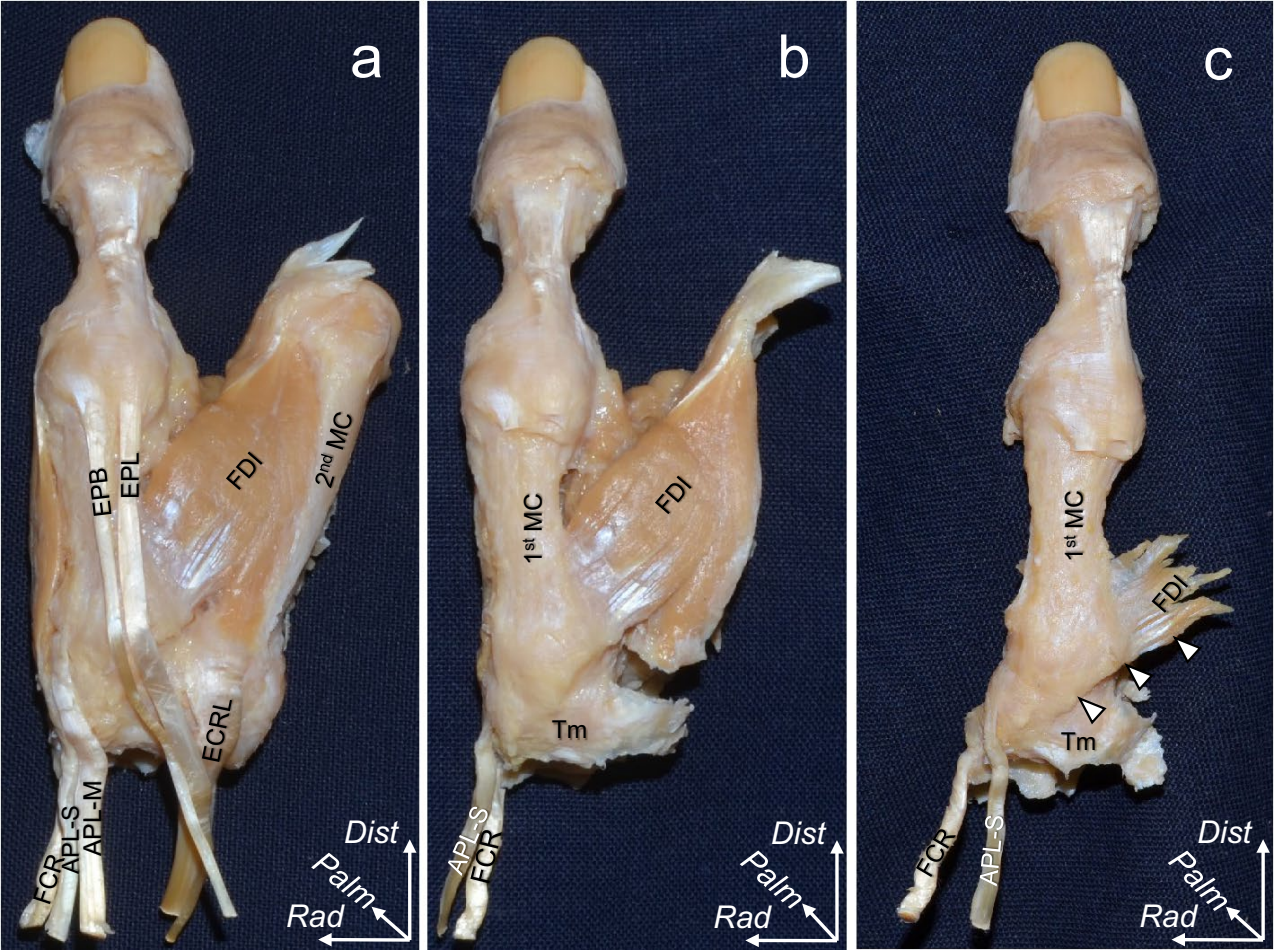
Macroscopic analysis of the dorsal aspect of the trapeziometacarpal joint. Photographs showing the layered relationships between the first dorsal interosseous (FDI) muscle and the joint capsule of the right thumb. (**a**) thumb and the second metacarpal bones (2nd MC) after removal of the skin and surrounding soft tissues. (**b**) the extensor pollicis brevis tendon (EPB), extensor pollicis longus tendon (EPL), extensor carpi radialis longus tendon (ECRL), 2nd MC, and abductor pollicis longus main tendon (APL-M) are removed from (**a**). (**c**): the muscular parts of the FDI are removed from (**b**), and the tendinous parts of the FDI and dorsal joint capsule (white arrowheads) are shown. APL-S, abductor pollicis longus–supernumerary tendon; FCR, flexor carpi radialis; Tm, trapezium; *Dist*, distal; *Rad*, radial; *Palm*, palmar.

On the dorsal aspect, the FDI originated from the proximal part of the 1st MC via a tendinous structure (Fig. 3a,b). After removal of the muscular parts of the FDI, the origin of the FDI aponeurosis was observed to extend proximally and intermingle with the dorsal TMC joint capsule to form the capsuloaponeurotic complex (Fig. 3c). These findings were observed in all specimens.

**Figure 4:**
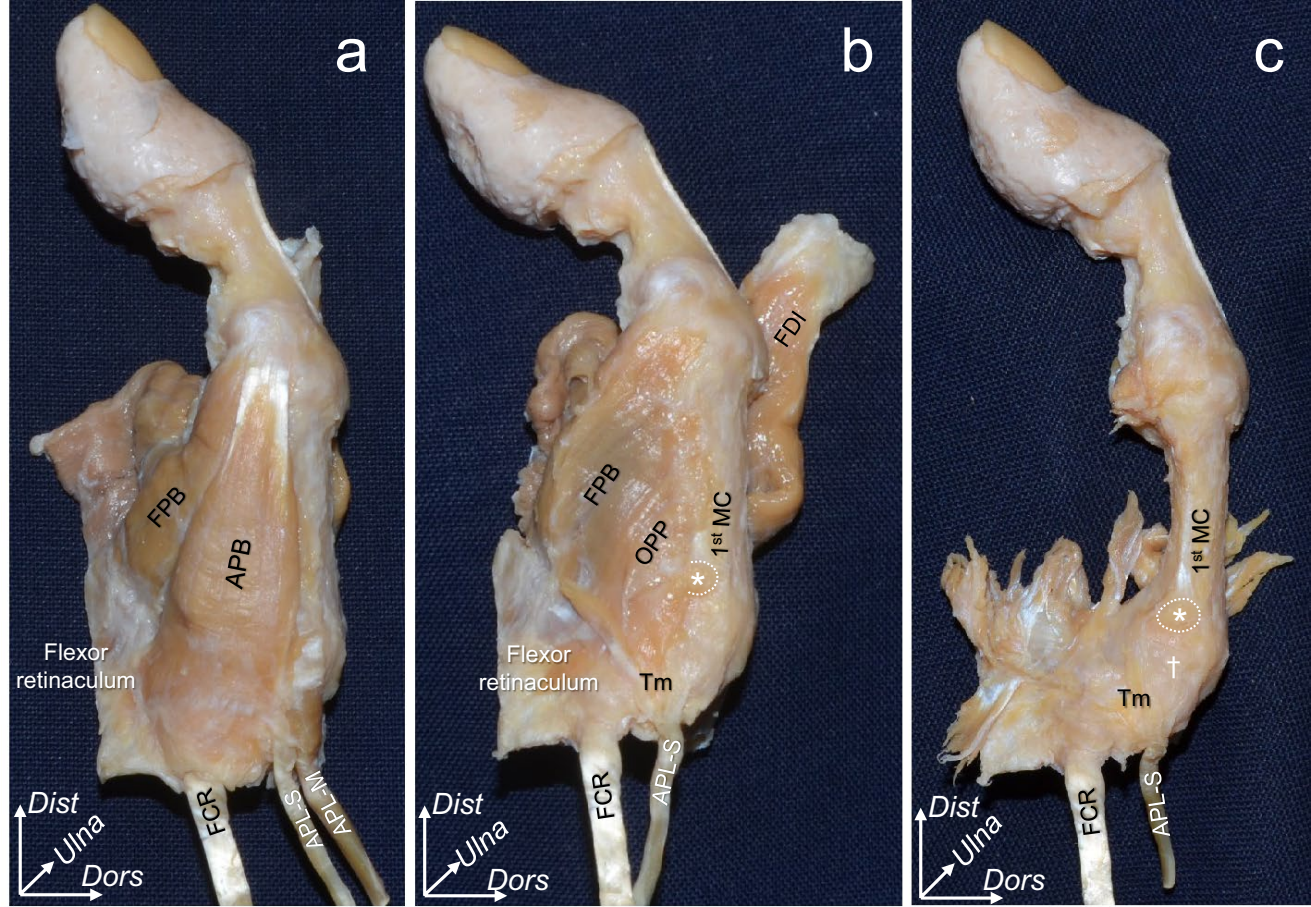
Macroscopic analysis of the radial aspect of the trapeziometacarpal joint. The photographs in Fig. 3 show the radial aspect (right thumb). The layered relationships among the abductor pollicis brevis (APB), opponens pollicis (OPP), flexor pollicis brevis (FPB), and joint capsule are indicated. (**a**) the radial side of Fig. 3b. (**b**) the APB and abductor pollicis longus main tendon (APL-M) are removed from (**a**). The attachment of the APL-M to the first metacarpal bone (1st MC) is indicated by an asterisk. (**c**) muscular parts of the FPB and OPP are removed from (**b**). The radial capsule in the deep layer of the FPB and OPP is indicated by a dagger. FCR, flexor carpi radialis; APL-S, abductor pollicis longus–supernumerary tendon; Tm, trapezium; *Dist*, distal; *Dors*, dorsal; *Ulna*, ulnar.

On the radial aspect, the abductor pollicis brevis (APB) originated from the flexor retinaculum, scaphoid, and trapezium and was inserted into the radial side of the proximal phalanx of the thumb (1st PP; Fig. 4a). The OPP and superficial flexor pollicis brevis (FPB) originated from the flexor retinaculum and the TMC joint capsule via muscular parts and were inserted into the 1st MC and 1st PP, respectively (Fig. 4b). The muscular border between the OPP and the superficial FPB could not be identified clearly. The abductor pollicis longus (APL) main tendon was attached to the radial base of the 1st MC, which corresponded to the brightly colored area in Fig. 2b, distal to the joint capsule attachment (Fig. 4b). After the removal of the muscular parts of the FPB and OPP, the radial joint capsule was observed to be thin (Fig. 4c). The APL supernumerary tendon was inserted into the TMC joint capsule in 16 out of 18 thumbs. The remaining two thumbs were from different cadavers.

**Figure 5:**
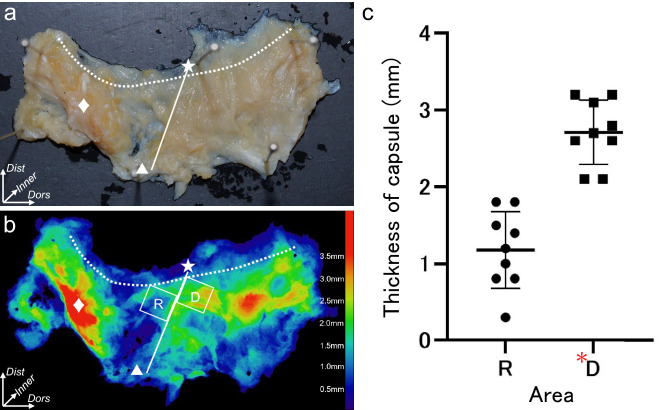
Distribution of the local thickness of the trapeziometacarpal joint capsule. (**a**) appearance of the trapeziometacarpal (TMC) joint capsule from the palmar aspect to the dorsal aspect (right thumb). The white star indicates attachment on the dorsoradial edge of the first metacarpal bones. The triangle indicates attachment of the APL supernumerary tendon. The rhomboid indicates the part corresponding with the radial roof of the flexor carpi radialis tendon groove (Dagger in Fig. 1c,d). The dotted line demonstrates the proximal edge of the attachment on the first metacarpal bone. The solid line indicates the borderline between the radial and dorsal aspects of the joint. (**b**) distribution of the local thickness of the joint capsule. The symbols are the same as in (**a**). Boxed regions R and D correspond to the radial and dorsal aspects of the TMC joint, respectively. The approximate thicknesses (mm) are shown by different colors in the color bar. c: scatter diagram showing the thicknesses of the R and D regions as shown in (**b**). The thicknesses are presented as means and standard deviations. Region D (red asterisk) is thicker than region R (paired t-test, P = 0.0000176). *Dist*, distal; *Dors*, dorsal.

The distribution analysis of the joint capsule thickness performed using micro-CT data revealed that the joint capsule on the dorsal aspect was brightly colored, which indicated that the dorsal aspect capsule was thicker than the radial aspect capsule (Fig. 5a,b). The mean capsule thickness was significantly greater on the dorsal aspect (2.7 ± 0.5 mm, **D** in Fig. 5b,c) than on the radial aspect (1.2 ± 0.5 mm, P = 0.000104, **R** in Fig. 5b,c).

### Histological analysis of the TMC joint

**Figure 6:**
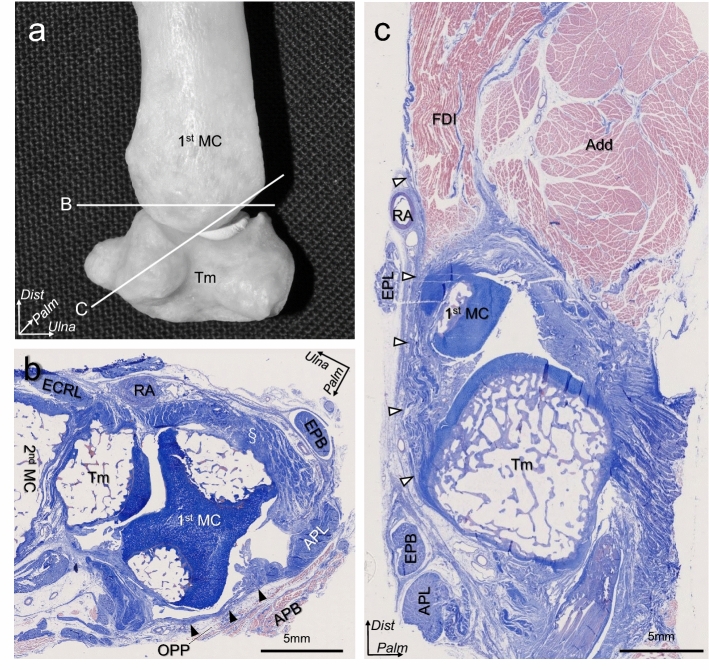
Histological analysis of the trapeziometacarpal joint. **(a**) Bone image showing the dissection levels (**b**) and (**c**) as white lines. (**b**) Axial section at the trapeziometacarpal joint level. Dorsal and radial joint capsules are shown as section marks and black arrowheads, respectively. (**c**): oblique sagittal section parallel to the fibrous orientation of the first dorsal interosseous (FDI) aponeurosis. The FDI aponeuroses are continuous with the dorsal joint capsule (white arrowheads). 2nd MC, second metacarpal bone; ECRL, extensor carpi radialis longus; EPB, extensor pollicis brevis; APL, abductor pollicis longus; OPP, opponens pollicis; FPB, flexor pollicis brevis; RA, radial artery; Add, adductor pollicis; EPL, extensor pollicis longus; *Ulna*, ulnar; *Palm*, palmar; *Dist*, distal; *Dors*, dorsal.

The axial section at the level of the TMC joint revealed that the joint capsule at the dorsal aspect was thick and densely stained (Fig. 6a,b). On the contrary, the radial aspect of the joint capsule was thin, underlying the muscular parts of the OPP and FPB. Oblique sagittal sections parallel to the fibrous orientation of the FDI aponeuroses demonstrated that the superficial FDI aponeurosis was continuous with the dorsal joint capsule and was attached to the dorsoradial tubercle of the trapezium via the fibrocartilage (Fig. 6c).

## Discussion

This study revealed that the dorsal aspect of the trapezium had a dorsoradial tubercle with cortical bone thickening. On the dorsal aspect, the aponeurosis of the FDI was intermingled with the dorsal TMC joint capsule, which was attached to the dorsoradial tubercle. On the radial aspect, a thin joint capsule underlay the muscular part of the OPP.

Previous studies described the tubercle on the dorsoradial aspect of the trapezium as the posterior tubercle^25^ and the dorsoradial tubercle^9,26–29^. Most reports have stated that the dorsoradial ligament is attached to these tubercles^9,26–29^. In the present study, we observed that the dorsal joint capsule was continuous with the FDI aponeurosis attached to the dorsoradial tubercle via the fibrocartilage. Furthermore, cortical bone thickening was observed in the dorsoradial tubercle. Because cortical bone thickening is assumed to reflect the tensile stresses applied to the bone^30–32^, the cortical bone thickening in the dorsoradial tubercle is in line with the function of the FDI to abduct the thumb, creating tensile stresses. We assumed that the FDI works as a dynamic stabilizer. There is a possibility that cortical bone thickening of the dorsoradial tubercle significantly varies with the frequency and amount of load during thumb abduction, which depends on antemortem occupations or activities of the specimen donors.

Previous studies noted two ligaments on the dorsal aspect of the TMC joint: one attached to the dorsoradial tubercle of the trapezium and the other running at its ulnar side^25,33,34^. Pieron^26^ defined these as the dorsoradial ligament (DRL) and the posterior oblique ligament (POL), respectively, and subsequent reports used these nomenclatures as well^1,9,28,35–38^. Furthermore, Hagert et al.^16^ and Ladd et al.^10^ added the dorsal central ligament (DCL), which is located and delineated between the DRL and POL. Some papers have described that these dorsal ligaments, including the DRL, POL, and DCL, are key for TMC joint stability as static stabilizers^8–10,16,26,34,36–38^. Regarding the anatomical relationships among the dorsal ligaments, periarticular muscles, and tendons, some studies have reported that the FDI arises from the dorsal ligaments^10,28^. In the present study, we demonstrated that the FDI aponeurosis was continuous with the dorsal joint capsule via the 1st MC. Boutan previously described an FDI aponeurosis directly inserted into the joint capsule^39^, which is compatible with our findings. Based on the results of the current study, the dorsal ligaments (including the DRL, DCL, and POL) could be interpreted as parts of the capsuloaponeurotic complex consisting of the FDI aponeurosis and joint capsule.

The joint capsule has been described in some reports on the radial aspect of the TMC joint^27,40^. Bettinger et al.^9^ and Pieron^26^ reported that the joint capsule was indistinguishable from the AOL. Based on previous studies, the structures on the radial aspect of the TMC joint have been described as thin and weak^9,26,27,40^. In the present study, we revealed that a thin radial joint capsule underlies the muscular parts of the OPP and FPB, which is compatible with previously reported anatomical findings.

Previous anatomical studies have reported that the supernumerary tendon of the APL is inserted into the APB, OPP, trapezium, and joint capsule^41–46^. In the present study, it was found to be inserted into the joint capsule, except in two thumbs; this incidence is relatively higher than that reported previously^42,44,47^. The APB and OPP covered the joint capsule, and the supernumerary tendon seemingly inserted and ended in the thenar muscle. However, the supernumerary tendon was confirmed to run deeply to insert into the joint capsule. There was a possibility that this portion of the supernumerary tendon (which ran deeply into the joint capsule) could be missed. Previous papers have controversially reported on the negative effects on the pathology of TMC osteoarthritis^47^ or the positive influences of the same on joint stability^42,44^. However, given that the radial aspect was only covered by a thin joint capsule, unlike the dorsal aspect that was covered by the thick capsuloaponeurotic complex, we speculated that the supernumerary tendon of the APL (which is inserted into the joint capsule) provided slight stability to the radial aspect of the TMC joint.

The results of this study have two clinical implications. First, this study may support the dynamic contribution of the surrounding muscles to TMC joint stability. Brand and Hollister^48^ had previously noted that the FDI helps to stabilize the TMC together with the OPP. Previous studies on both cadaveric and living specimens have described that the FDI contributes to joint stability^11–13^; this is compatible with our finding that the FDI and the joint capsule formed the capsuloaponeurotic complex. The contribution of OPP in cadaveric specimens has been reported^13^; however, it has not yet been verified in living specimens. Based on the anatomical findings of the present study, the FDI could provide tension to stabilize the dorsal aspect of the TMC joint, as described previously. Meanwhile, no fibrous structures were observed, which were assumed to stabilize the radial aspect of the TMC joint, except for the thin joint capsule. In other words, OPP muscular activation, which covers the radial aspect of the TMC joint, is assumed as essential for joint stabilization. Previously, Edmunds^49^ described that a dynamic force couple is formed by the OPP, volar beak, dorsal ligament complex, and articular congruence. This description could support our speculation about the OPP function. To verify the involvement of OPP in joint stabilization in living specimens, further studies using various modalities (such as in vivo imaging, computational modelling, or electromyography) are necessary.

Second, based on our anatomical findings, we could assume functional relationships between OPP and FPB. In this study, the muscular parts of the OPP and superficial FPB had no clear borders, similar to descriptions in previous papers^48,50^. Furthermore, Brand and Hollister^48^ had noted that the function of the FPB is continuous with that of the OPP. Therefore, dysfunction in one may lead to dysfunction in the other. As such, this might explain the coexistence of the TMC joint deformity and metacarpophalangeal joint hyperextension of the thumb as a pathology.

This study has some limitations. First, it was purely anatomical in nature. The dynamic contribution of the surrounding muscles to the TMC joint instability was not evaluated. Therefore, prospective studies with additional imaging evaluations and clinical cases are necessary to validate our findings. Second, this study was limited to Japanese specimens; the insertion sites of the supernumerary tendon could vary among different ethnicities. Third, the methods for measuring cortical bone and TMC joint capsule thicknesses were cumbersome; therefore, the results might vary. To validate these methods, we assessed intraclass correlation coefficients (ICCs). Because the ICC scores in our study were 0.85 and 0.995, we believe that these methods were reproducible.

In conclusion, on the dorsal aspect of the TMC joint, the capsuloaponeurotic complex (consisting of the FDI aponeurosis and joint capsule) is attached to the dorsoradial tubercle with cortical bone thickening. On the radial aspect, the thin joint capsule underlies the OPP muscles without fibrous structures. Our findings may help to elucidate the pathogenesis of TMC joint instability and contribute to the appropriate management of TMC osteoarthritis.

## Methods

### Preparation of cadaveric specimens and micro-CT imaging

We used a total of 42 thumbs (21 right and 21 left) from 27 Japanese cadavers; these comprised 8 male and 19 female donors. The mean age of the donors (and standard deviation) at the time of death was 83.2 ± 12.8 years (range, 52–99 years). Before their death, all donors had declared that their bodies would be donated to the Department of Anatomy of Tokyo Medical and Dental University for educational purposes. Then, we explained the purpose and methods of using the donors’ corpses and informed consent was obtained. Furthermore, after their death, we also explained to the next of kin that the informed consent had been obtained. Our study is in compliance with the Japanese law named “Act on Body Donation for Medical and Dental Education.” The study design was approved by the Medical Research Ethics Committee of Tokyo Medical and Dental University (#M2019-264). All procedures were performed in accordance with the Japanese “Ethical Guidelines for Medical and Health Research Involving Human Subjects”.

All cadaver specimens were fixed in 8% formalin and were preserved in 30% ethanol. We cut them proximally at the level of the wrist and medially at the level between the second and the third fingers using a diamond band pathology saw (EXAKT 312; EXAKT Advanced Technologies GmbH, Norderstedt, Germany). After removal of the skin and subcutaneous tissues, we examined the osseous configuration of all specimens using micro-CT with a 200-μm resolution (inspeXio SMX-100CT; Shimadzu Corp., Kyoto, Japan); all images were analyzed using the ImageJ software (version 1.52; National Institutes of Health, Bethesda, MD, USA). We excluded 17 specimens with advanced TMC osteoarthritis (Eaton and Glickel classification stages III and IV [determined using CT])^51^. Of the remaining 25 specimens (13 right and 12 left), 1, 18, and 6 were randomly assigned for chemically debrided bone, macroscopic, and histological analyses, respectively.

### Bone morphology and cortical bone thickness mapping of the 1st MC and the trapezium

To visualize the bone morphology of the dorsal and radial aspects of the 1st MC and trapezium, three-dimensional (3D) images were reconstructed using 8-bit images of the 25 specimens obtained using the ImageJ software, as described previously (Fig. 1a,c)^30^. Furthermore, to confirm the bone morphology in images obtained by ImageJ without dissection artifacts, we chemically removed the soft tissues of one thumb using 0.8% sodium hydroxide solution (Wako Pure Chemical Industries, Osaka, Japan; Fig. 1b,d).

To visualize the cortical bone thickness distribution on the dorsal and radial aspects of the 1st MC and trapezium, BoneJ (an ImageJ plugin) was used^31^. BoneJ defined thickness at a point by measuring the diameter of the largest sphere that fit within the structure of interest (Fig. 2)^30,32,52^. The cortical bone thicknesses of the 1st MC and trapezium were mapped onto 3D images; thicker areas of cortical bone were represented by brighter colors.

To compare the cortical bone thickness between the dorsoradial tubercle and other areas, we used axial images at the level of the proximal one-third of the trapezium. We defined a 3.0 × 8.0 mm rectangle as the smallest rectangle, which fully covered the cortical bone of the dorsoradial tubercles in all specimens, as a region of interest. First, we set the rectangle at the dorsal aspect as DR, which was supposed to include the tip and radial and ulnar foundations of the dorsoradial tubercle, and be parallel to the cortical bone surface. Next, other rectangles were set to contact with the ulnar and radial sides of the DR (DU and R, respectively), which were supposed to be parallel to the cortical bone surface. We calculated and compared the mean bone thickness (with the standard deviation) in the quadrants. The observer randomly repeated the measurements twice to determine intra-observer reproducibility^21,23^.

### Macroscopic analysis: relationship between the TMC joint capsule and the adjoining muscles

Eighteen thumbs were analyzed macroscopically. Initially, we removed the skin, subcutaneous tissues, flexor tendons, and median nerve in the carpal tunnel. The index finger phalanges, radius, and carpal bones (except for the trapezium and trapezoid) were also discarded (Figs. 3a and 4a). Next, we removed the extensor tendons, second metacarpal, trapezoid, APB, and APL (Figs. 3b and 4b). To reveal the relationships among the tendinous parts of the FDI, OPP, FPB, and TMC joint capsule, we carefully removed parts of these muscles as well (Figs. 3c and 4c).

### Evaluation of the thickness of the TMC joint capsule

Of the 18 specimens analyzed macroscopically, nine were randomly assigned for micro-CT evaluation of the joint capsule thickness. The entire capsule was sectioned at the ulnar aspect, detached from the 1st MC and trapezium (Fig. 5a), and analyzed using micro-CT with a resolution of 200 µm (Fig. 5b)^53,54^. Images of the joint capsule were reconstructed to be parallel to the flattened joint capsule. A real image was created by the stacked images; these were binarized images for all serial frames extracted from the micro-CT images. The thickness of the joint capsule was determined using the slice unit number and length. To visualize the distribution of the capsule thickness, we created a color look-up table. We compared the mean capsule thickness between the radial and dorsal aspects using 5-mm square regions (Fig. 5b). First, we defined the line from the dorsal edge of the APL attachment of the 1st MC to the dorsoradial edge of the trapezium as the border between the radial and dorsal aspects. Next, the dorsal and radial regions were set along both aspects of the borderline. Furthermore, the regions were set at the most distal part so as not to overlap the capsular attachment on the 1st MC.

### Histological analysis: relationship between the TMC joint capsule and its superficial muscles

We performed histological examinations of the TMC joints in six thumbs that were selected randomly. Using the EXAKT 312 saw, we obtained three blocks perpendicular to the TMC joint from three thumbs (Fig. 6a). From the remaining three thumbs, we obtained blocks parallel to the tendinous fibers of the FDI. The obtained blocks were decalcified for 1 week with a Plank–Rychlo solution (AlCl_3_:6H_2_O [126.7 g/L], HCl [85.0 ml/L], and HCOOH [50.0 ml/L])^55^ and were dehydrated. After dehydration, the blocks were embedded in a paraffin solution and sectioned serially (thickness, 2.5 μm). The sections were then stained using Masson’s trichrome staining protocol.

### Statistical analysis

Statistical tests were performed using the statistical software “Easy R for Windows” (version 4.0.3; R Development Core Team), which is based on R and R commander. The cortical bone thicknesses on the axial images of the trapezium were compared among the DR, DU, and R groups using a repeated-measures analysis of variance. When significant differences among the areas were evident, comparisons were made among the DR, DU, and R groups using the Bonferroni *post-hoc* test. The thickness of the capsule was compared between the dorsal and radial aspects using a paired t-test. Significance was set at P < 0.05. ICCs were assessed using an inter-rater reliability analysis. The qualitative cutoffs of the ICCs were as follows: poor, < 0.5; moderate, 0.50–0.75; good, 0.75–0.9; and excellent, > 0.9^56^. For measurement of the cortical bone and capsule thicknesses, the ICC scores in our study were 0.85 (95% confidence interval [CI], 0.77–0.90) and 0.995 [95% CI, 0.989–0.998], respectively.

## Supplementary Information

Supplementary Table S1.

## Data Availability

The datasets used and/or analyzed during the present study are available from the corresponding author upon reasonable request.
