# Supplementary material for: Anatomical study for elucidating the stabilization mechanism in the trapeziometacarpal joint
Source: Sci Rep. 2022 Dec 1;12:20790. doi: 10.1038/s41598-022-25355-3 (PMC9715720; doi:10.1038/s41598-022-25355-3)
Supplement: Supplementary file 1 — Supplementary Table S1. [file 41598_2022_25355_MOESM1_ESM.docx]

**Anatomical study for elucidating the stabilization mechanism in the trapeziometacarpal joint**

Mio Norose, MD^1,2^, Akimoto Nimura, MD, PhD^3^*, Masahiro Tsutsumi, PhD^1,4^,

Koji Fujita, MD, PhD^3^, Atsushi Okawa, MD^2^, PhD, and Keiichi Akita, MD, PhD^1^

**Supplementary Table S1.** Cortical thickness of the trapezium in the subgroups.

| Age or sex | Numbers | Regions | | |
| --- | --- | --- | --- | --- |
|  |  | R (mm) | DR (mm) | DU (mm) |
| < 85 years | 12 | 0.2±0.0 | 0.4±0.1 | 0.2±0.1 |
| (P value vs. DR) |  | 0.0065* | NA | 0.0063* |
| ≥ 85 years | 13 | 0.2±0.1 | 0.4±0.2 | 0.2±0.1 |
| (P value vs. DR) |  | 0.0065* | NA | 0.0063* |
| Male | 7 | 0.2±0.1 | 0.4±0.2 | 0.3±0.2 |
| (P value vs. DR) |  | 0.083 | NA | 0.08 |
| Female | 18 | 0.2±0.1 | 0.3±0.1 | 0.2±0.1 |
| (P value vs. DR) |  | 0.00011* | NA | 0.00002* |
| Total | 25 | 0.2±0.1 | 0.4±0.2 | 0.2±0.1 |
| (P value vs. DR) |  | 0.000019* | NA | 0.00000092* |

A repeated-measures analysis of variance and a paired t-test were performed.

The significance level was set at p<0.05 (asterisk).

The locations of the measurements are demonstrated in Figure 2c.

Cortical thickness is presented as mean±standard deviation.
